# Piscine orthoreovirus (PRV) replicates in Atlantic salmon (*Salmo salar* L.) erythrocytes ex vivo

**DOI:** 10.1186/s13567-015-0154-7

**Published:** 2015-03-06

**Authors:** Øystein Wessel, Christel Moræus Olsen, Espen Rimstad, Maria Krudtaa Dahle

**Affiliations:** Department of Food Safety and Infection Biology, Norwegian University of Life Sciences, Oslo, Norway; Section of Immunology, Norwegian Veterinary Institute, Oslo, Norway

## Abstract

Piscine orthoreovirus (PRV) is a reovirus that has predominantly been detected in Atlantic salmon (*Salmo salar* L*.*). PRV is associated with heart and skeletal muscle inflammation (HSMI) in farmed Atlantic salmon, and recently erythrocytes were identified as major target cells. The study of PRV replication and pathogenesis of the infection has been impeded by the inability to propagate PRV in vitro. In this study we developed an ex vivo cultivation system for PRV in Atlantic salmon erythrocytes. PRV was successfully passaged to naïve erythrocytes using lysates of blood cells from infected salmon. During cultivation a significant increase in viral load was observed by RT-qPCR and flow cytometry, which coincided with the formation of cytoplasmic inclusions. The inclusions resembled viral factories and contained both PRV protein and dsRNA. In addition, the erythrocytes generated an antiviral immune gene activation after PRV infection, with significant up-regulation of IFN-α, RIG-I, Mx and PKR transcripts. Supernatants from the first passage successfully transmitted virus to naïve erythrocytes. This study demonstrates that PRV replicates in Atlantic salmon erythrocytes ex vivo. The ex vivo infection model closely reflects the situation in vivo and can be used to study the infection and replication mechanisms of PRV, as well as the antiviral immune responses of salmonid erythrocytes.

## Introduction

Piscine orthoreovirus (PRV) has a double-stranded RNA (dsRNA) genome and belongs to the family *Reoviridae* [1]. PRV primarily infects Atlantic salmon (*Salmo salar* L.) and is associated with heart and skeletal muscle inflammation (HSMI), an important disease of farmed Atlantic salmon in Norway [1,2]. The virus is ubiquitously present in both diseased and apparently healthy individuals [3]. PRV is widespread in farmed salmonids in Europe, North and South America and present in several species of wild salmonids [4-6]. However, PRV has only been associated with disease in farmed Atlantic salmon. The study of viral replication and pathogenesis of the infection has been hindered by the inability to propagate PRV efficiently in cell cultures.

Phylogenetically PRV branches off the common root of the genera *Orthoreovirus* and *Aquareovirus,* but clusters more closely with the orthoreoviruses, although it is a fish virus [7-9]. Among orthoreoviruses, mammalian orthoreovirus (MRV) has been studied extensively and used as a model system for investigations of virus-host interactions, which has generated a wealth of information regarding replication cycle, protein functions and virion structure [10]. Even if nucleotide and protein sequence identities between the mammalian orthoreoviruses and PRV are low, important structural motifs and key amino acid are conserved in homologous proteins [7,9]. Currently PRV studies have been restricted to bioinformatic analyses [4,9], RT-qPCR screenings, field epidemiology [3,6] or laborious and costly in vivo challenge studies [11,12]. There have been a number of attempts to culture PRV in vitro. Cytopathic effects (CPE) have been observed in several cell lines including epithelioma papulosum cyprini (EPC), fathead minnow (FHM) and GF-1 derived from orange-spotted grouper [11,13]. However, increase in PRV titer has not been shown in any of these cell lines. An efficient in vitro cultivation system would be a highly desired tool of PRV research.

The association between PRV and HSMI has been strengthened in recent studies. The high viral RNA loads in the heart and the presence of viral antigens in cardiomyocytes correlate with development of the cardiac lesions observed in HSMI [11,14]. However, an explanation for high viral load in both farmed and wild salmon without HSMI lesions is lacking [3,6], and in Canada PRV is present without HSMI being diagnosed [5]. This underlines the importance to study the PRV pathogenesis. We have recently demonstrated high viral loads in blood prior to PRV presence in cardiomyocytes and shown that red blood cells (RBC) are major target cells for PRV [12,14]. At peak infection phase more than 50% PRV positive erythrocytes were detected in individual fish. Despite the massive PRV infection of RBC, clinical anemia is not associated with HSMI or a PRV infection [15]. The study of PRV infection in erythrocytes will be important to understand the pathogenesis of PRV infection and its implications on Atlantic salmon health.

In MRV infected cells, viral-inclusions are present in distinct areas of the cell during replication; these are recognized as viral factories [16]. The cytoplasmic inclusions observed in PRV infected RBC resemble the globular viral factories of MRV type 3 prototype strain Dearing (T3D) [17,18]. The PRV inclusions contain reovirus-like particles as demonstrated by transmission electron microscopy (TEM) [12]. Inclusions and virus-like particles have previously been reported in erythrocytes of salmonid fish, suggesting that such infections are common [19-22]. The most commonly reported is erythrocytic inclusions body syndrome (EIBS) [23]. However, these findings have not been associated with any specific viral agent. The reovirus-like particles and intraerythrocytic inclusions observed in PRV infected RBC may resemble some of those described as EIBS [24-26]. Piscine erythrocytes, in contrast to mammalian erythrocytes, are nucleated cells containing organelles and capable of protein production [27,28]. Therefore, piscine erythrocytes are potentially able to support viral replication.

Recent studies have indicated that piscine erythrocytes may have immune functions [29]. Rainbow trout erythrocytes stimulated with Poly(I:C) respond by expression of genes encoding pathogen receptors (including toll-like receptors (TLRs)), interferon alpha (IFN-α) and immune cell recruiting mediators like CC chemokine (CCL) 4 [30]. Erythrocytes from Atlantic salmon have been shown to respond to infectious salmon anemia virus (ISAV) by production of IFN-α [31]. An important trigger of interferon production is recognition of dsRNA, either in endosomal compartments by the transmembrane receptor TLR3, or in the cytoplasm by RNA helicases like Retinoic-acid inducible gene (RIG)-I and Melanoma differentiation-associated protein (MDA)-5 [32,33]. In addition, cytosolic dsRNA bind dsRNA-activated kinases like PKR and PKZ and induce partial translational shutdown [34,35]. In order to bypass these antiviral responses, dsRNA viruses have developed counter-mechanisms. These include avoiding the exposure of the dsRNA genome by performing transcription and replication within the viral core, masking the viral RNA by dsRNA-binding viral proteins, inhibiting the dsRNA signaling pathway, or by producing non-polyadenylated viral mRNA which can omit the translational shutdown [36]. All these mechanisms have been demonstrated for MRV [37,38], and it is likely, due to structural similarities, that PRV counteracts antiviral mechanisms in similar ways. Recently, it was shown that PRV protein ơ3 binds dsRNA in a sequence independent manner, thus sharing this function with MRV ơ3 [39].

Through in vivo challenge experiments we have recently found that RBC is an important target cell for PRV infection in salmon [12], and we hypothesized that PRV can replicate in Atlantic salmon erythrocytes cultures ex vivo. In this study, supernatant from lysed blood cells collected from in vivo PRV infected salmon were passaged to RBC isolated from naïve salmon and cultured ex vivo. The viral propagation was monitored by RT-qPCR, flow cytometry, and immunofluorescence microscopy. The ability of the recipient erythrocytes to elicit an innate antiviral response was also assessed. Finally, a supernatant from the first passage was used to passage PRV to naïve RBC.

## Materials and methods

### Blood samples

Heparinized blood (0.5 mL) was collected from the caudal vein of six naïve Atlantic salmon (F1-F6). The fish had an average weight of 40 g and originated from a naïve population kept in a fresh water tank at VESO aquatic research facility (Vikan, Norway). The fish were anesthetized prior to sampling by bath immersion (2–5 min) in benzocaine chloride (0.5 g/10 L water) and euthanized after sampling using benzocaine chloride (1 g/5 L water) for 5 min. The RBC were isolated from the blood samples as described below and used as recipient erythrocytes in the first passage. Three weeks later heparinized blood samples were collected from six fish (F11-F16) of the same fish population. RBC were isolated and used as recipient erythrocytes in the second passage.

### Isolation of naïve RBC

We diluted the heparinized blood 1:15 in phosphate-buffered saline (PBS) and isolated the RBC using a Percoll gradient as previously described [12]. The cells were counted using Countess (Invitrogen, Eugene, Oregon, USA) and resuspended to a concentration of 3 × 10^7^ cells/mL in Leibovitz’s L15 medium (Life Technologies, Carlsbad, CA, USA) supplemented with fetal calf serum (2%) and gentamicin (50 μg/mL). All recipient RBCs (first passage F1-F6; second passage F11-F16) were confirmed PRV-negative by RT-qPCR and flow cytometry before initiation of the study (described below).

### Preparation of inoculums

The PRV inoculum was prepared from a batch of pooled heparinized blood samples collected 6 weeks post challenge (wpc) from cohabitant fish of a PRV challenge experiment (VESO, Vikan, Norway). We centrifuged the pooled blood sample at 2000 *g* for 10 min at 4 °C, removed the plasma and diluted the blood pellet 1:10 in L15 with gentamicin (50 μg/mL). The diluted blood was then sonicated on ice by 8 × 10 s pulses at 25 Hz with 30 s rest in between, and the lysed blood was centrifuged at 2000 *g* for 5 min at 4 °C to remove cellular debris. The resulting supernatant was used as PRV inoculum in the first passage. Similarly, a negative control inoculum was prepared as described above, using heparinized blood collected from naïve fish. The control inoculum was confirmed PRV negative by RT-qPCR. The PRV inoculum used had a Ct-value of 22.3.

### Ex vivo setup; 1^st^ passage

In the first PRV passage, RBC isolated from six naïve fish were inoculated and cultured ex vivo for 21 days. The culture experiment was performed in 24-deepwell microtiter plates with pyramidal bottom (AB Ninolab, Stockholm, Sweden) to allow easy centrifugation and washing of the cells. The plates were closed using Mikro-Flask sandwich cover and clamp system by Duetz (Applikon, Foster City, CA, USA) to minimize evaporation and prevent well to well contamination. Incubation was performed at 15 °C in an Ecotron incubation shaker (Infors HT, Basel Switzerland) shaken at 225 rpm to ensure that the RBC was kept in a homogenous suspension.

The cultivation setup included four identical PRV infected plates and four control plates that were sequentially harvested at 1, 7, 14 and 21 days post infection (dpi). On each plate, RBC isolated from fish F1-F6 was plated at 3 × 10^7^ RBC per well in 1 mL medium. The wells were subsequently inoculated with 100 μL of either the PRV-infected inoculate or the control inoculate described above. Following 24 h of incubation the plates were centrifuged at 500 *g* for 5 min at 4 °C to remove the supernatant and washed in Leibovitz’s L15 medium (Life Technologies). Finally, the RBC was resuspended to a final concentration of 10 × 10^6^ RBC/mL in 3 mL Leibovitz’s L15 medium (Life Technologies) supplemented with fetal calf serum (2%) and gentamicin (50 μg/mL). The 1 dpi samples were sampled immediately after resuspension and the remaining plates were incubated until harvested at 7, 14 and 21 dpi respectively. The 0 dpi samples were collected prior to inoculation.

At each time point a sample of 2 mL (20 × 10^6^ RBC) was collected from each well for RNA isolation and subsequent RT-qPCR analysis to assess the viral load in the RBC and the supernatant. In addition, the immune response of infected RBC was assayed. The remaining 1 mL (10 × 10^6^ RBC) was used for PRV detection by flow cytometry analysis and immunofluorescence/confocal microscopy to study viral inclusions. The number of cells were counted using Countess (Invitrogen) in order to determine the amount of cell lysis. Details for all analyses are described below.

### Ex vivo setup; 2^nd^ passage

In the second PRV passage, RBC isolated from six fish were infected with supernatant from the first passage and cultured ex vivo for 21 days. A pool of supernatant was prepared from the 21 dpi samples of the first passage after centrifugation at 1000 *g* for 5 min at 4 °C and used as inoculum.

The cultivation setup included two identical PRV infected plates that were sequentially harvested at 14 and 21 dpi. On each plate, RBC isolated from fish F11-F16 was plated at 3 × 10^7^ RBC per well in 1 mL media as for the first passage. The cells were subsequently inoculated with 100 μL of a 10-fold dilution of the supernatant from the first passage. The RBC were incubated for 24 h, centrifuged, washed and resuspended in growth medium to a final concentration of 10 × 10^6^ RBC/mL. The plates were cultured under identical conditions as those of the first passage and harvested at 14 and 21 dpi. The 0 dpi samples had been collected prior to inoculation.

At each time point a sample of 2 mL (20 × 10^6^ RBC) was collected from each well for RNA isolation and RT-qPCR analysis to detect PRV in the RBC fraction and 1 mL was used for flow cytometry analysis and immunofluorescence microscopy. Details for the analyses are described below.

### RNA isolation

The 2 × 10^6^ cells (2 mL) harvested from each culture were pelleted at 1000 *g* for 5 min at 4 °C and total RNA was isolated from the RBC pellet and the supernatant. The RBC pellet was homogenized in Isol-RNA Lysis Reagent (5 PRIME, Hilden, Germany) using 5 mm steel beads and TissueLyser II (Qiagen) for 2 × 5 min at 25 Hz. After addition of chloroform and centrifugation, the aqueous phase was collected and proceeded with automated RNA isolation using RNeasy Mini QIAcube Kit (Qiagen) as described by the manufacturer. For the supernatant samples, a combination of Trizol LS (Invitrogen) and RNeasy Mini spin column (Qiagen) was used. Briefly, 100 μL supernatant was mixed and incubated with Trizol LS before adding chloroform, separating the phases by centrifugation, and then proceeding with the RNeasy Mini spin column as recommended by the manufacturer, eluting isolated RNA in 50 μL RNase-free water. RNA was quantified using a NanoDrop ND-1000 spectrophotometer (Thermo Fisher Scientific, Wilmington, DE, USA).

### RT-qPCR; PRV detection

The Qiagen OneStep kit (Qiagen) was used for RT-qPCR. The input was 100 ng (5 μL of 20 ng/uL) total RNA from RBC per reaction and 5 μL purified RNA solution from the cell free samples, i.e. supernatants and inoculums. RT-qPCR targeted PRV segment S1 using the following conditions: 400 nM primer, 300 nM probe, 400 nM dNTPs, 1.26 mM MgCl2, 1:100 RNase Out (Invitrogen) and 1 × ROX reference dye with the following cycle parameters: 30 min at 50 °C, 15 min at 94 °C, 35 cycles of 94 °C/15 s, 54 °C/30 s and 72 °C/15 s in a Mx3005P (Stratagene, La Jolla, CA, USA). The samples were run in duplicate, and a sample was defined as positive if both parallel samples had a Ct < 35. Primers and probe are listed in Table 1.

**Table 1:** **Primers and probe used in this study**

**Primer/probes**	**Primer sequence (5’ → 3’)**	**Accession #**
ơ3 S1-659 Fwd	TGCGTCCTGCGTATGGCACC	GU994022
ơ3 S1-801 Rev	GGCTGGCATGCCCGAATAGCA	
ơ3 S1-693 Probe	*FAM-*ATCACAACGCCTACCT–*MGBNFQ*	
IFNα Fwd	ACTGAAACGCTACTTCAAGAAGTTGA	AY216595
IFNα Rev	GCAGATGACGTTTTGTCTCTTTCCT	
Mx Fwd	GATGCTGCACCTCAAGTCCTATTA	BT043721.1
Mx Rev	CACCAGGTAGCGGATCACCAT	
RIG-I Fwd	ACGCCTTGAAGAGCTGGATA	FN178459
RIG-I Rev	CTGGCTGGACTTGTGTCCTC	
PKR Fwd	CAGGATGCAACACCATCATC	EF523422.1
PKR Rev	GGTCTTGACCGGTGACATCT	

The quencher and reporter dye of the probes are in italic. Accession numbers are shown in the right column.

### qPCR; Antiviral response

Reverse transcription was performed using 200 ng RNA per sample, and a mixed input from representative samples was used to prepare a seven point concentration standard curve. The RNA was denaturated at 95 °C for 5 min prior to cDNA synthesis, using the Quantitect reverse transcription kit with integrated genomic DNA removal (Qiagen). Quantitative PCR (qPCR) was run in duplicate on cDNA corresponding to 10 ng RNA input. The antiviral response gene expression analysis was performed using 2 × Maxima SYBR Green qPCR Master Mix (Thermo Fisher scientific, Waltham, MA, USA) and 500 nM specific primers targeting Atlantic salmon IFNα, myxovirus resistance (Mx), RIG-I and PKR respectively. The assays were run 40 cycles of 94 °C/15 s, 60 °C/30 s in a Mx3005P device (Stratagene). Primers used are listed in Table 1.

### Flow cytometry

RBC sampled during the first and second passage were screened for PRV by flow cytometry. The RBC were stained intracellularly using a rabbit polyclonal antibody against PRV putative outer capsid protein ơ1 (Anti-ơ1, #K275) as previously described [12]. The corresponding zero serum (Anti-σ1 Zero #K275) [14] was used as negative control serum. The cells were analyzed on a Gallios Flow Cytometer (Beckman Coulter, Miami, FL, USA) counting 30 000 cells per sample. The data were analyzed using the Kaluza software (Becton Dickinson). The forward/side gate was set to adjust for variation in background staining. The arithmetic mean fluorescence intensity (MFI) was calculated for all PRV infected (green) and control (grey) samples at each time point, in addition to the ΔMFI. The differences were calculated statistically using Wilcoxon matched pairs signed rank test due to the small sample size (*n* = 6).

### Immunofluorescence microscopy

The PRV σ1 stained RBC used in flow cytometry were also assayed by immunofluorescence microscopy. The nuclei were stained with Hoechst 33342 (Invitrogen) before they were transferred to Countess chamber slides (Invitrogen). Photographs were taken by an inverted fluorescence microscope (Olympus IX81), at 20× and 40× magnification.

### Confocal microscopy

Smears of RBC were dried before fixation for 5 min in ice cold methanol. Slides were rehydrated in PBS and blocked in PBS with 5% skimmed milk before sequentially staining with a mouse monoclonal antibody against dsRNA (J2; 1:200) (Scicons, Hungary) and rabbit anti-ơ1 (1:750) with washing in-between. Secondary antibody; goat anti-mouse Alexa Fluor 488 and goat anti-rabbit Alexa Fluor 594 (1:1000) were obtained from Molecular Probes (Invitrogen). The nucleus was counterstained with Hoechst 33342 (Invitrogen) and coverslips were mounted with Fluoroshield (Sigma-Aldrich). The images were generated on an inverted Zeiss LSM 710 confocal laser scanning microscope (CLSM) (Carl Zeiss, Oberkochen, Germany), using lasers of 405 nm, 488 nm and 594 nm to excite the respective stains. In post-production, the original color of dsRNA (Alexa Flour 488) and PRV-ơ1 protein (Alexa Flour 594) was switched to match the immunofluorescence pictures, in which PRV-ơ1 protein was colored green.

### Data analysis

The PRV RT-qPCR results from 1 dpi were compared to paired samples collected at 7, 14 and 21 dpi in the first. For the immune gene analysis of IFNα, Mx, RIG-I and PKR the fold increase at 1, 7, 14 and 21 dpi was calculated, assigning the qPCR results from the uninfected paired samples as 1. In the second passage, the results from the undiluted and the 10 fold diluted inoculum was compared at each time point. The differences were analyzed statistically using Wilcoxon matched pairs signed rank test due to the small sample size (*n* = 6). All statistical analysis described were performed with GraphPad Prism (GraphPad Software inc., USA) and *p*-values of *p* ≤ 0.05 were considered as significant.

## Results

A blood cell lysate prepared from PRV infected fish was used as inoculum in the first passage of PRV to naïve erythrocytes. The inoculum had Ct value of 22.3. The recipient naïve RBC were confirmed negative for PRV by both RT-qPCR and flow cytometry before onset of the study and they were cultured ex vivo for 21 dpi.

### Increased PRV RNA shown by RT-qPCR

**Figure 1:**
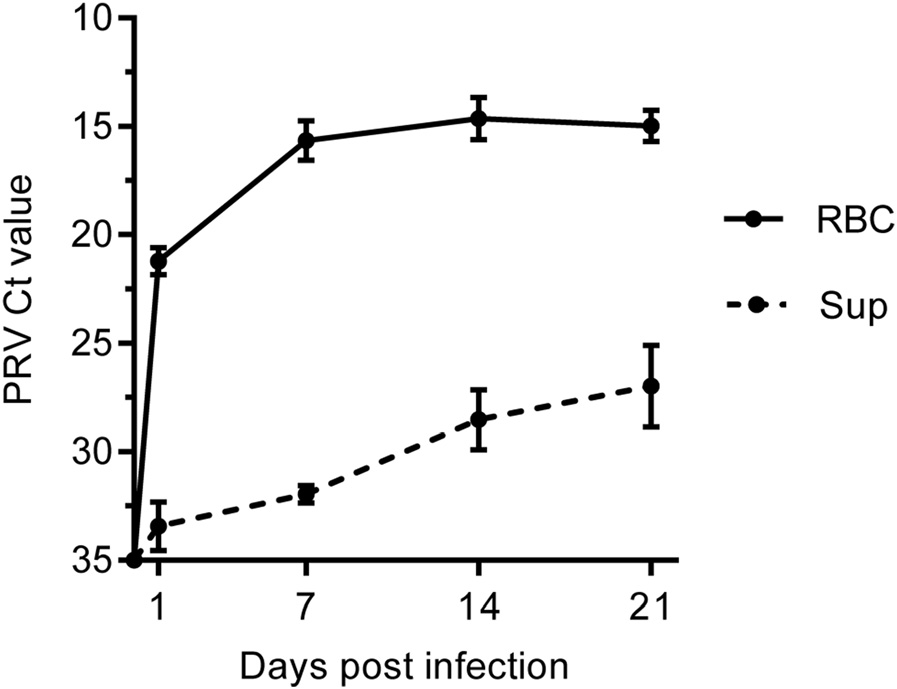
**PRV load in 1** ^**st**^ **passage of culturing.** Mean PRV Ct value with SD (*n* = 6) as detected by RT-qPCR in red blood cells (RBC) and supernatant (Sup) at 1, 7, 14 and 21 days post infection.

Following inoculation with PRV containing lysate, there was an increase in the PRV titer in both RBC and supernatant during the first passage (Figure 1). At 1 dpi, after removal of the inoculum and washing of the cells, the mean Ct value was 21.2 ± 0.6 in the RBC fraction. A significant increase in viral load was observed from 1 dpi to 14 dpi when the Ct was 14.6 ± 0.9 (*p* < 0.05), and it stayed on this level until the end of the first passage at 21 dpi (Ct 14.9 ± 0.7). Similarly, in the supernatant there was a significant increase in mean Ct-value from 1 dpi (Ct 33.4 ± 1.1) to 21 dpi (Ct 27.0 ± 1.8) (*p* < 0.05).

### Increased PRV protein shown by flow cytometry

**Figure 2:**
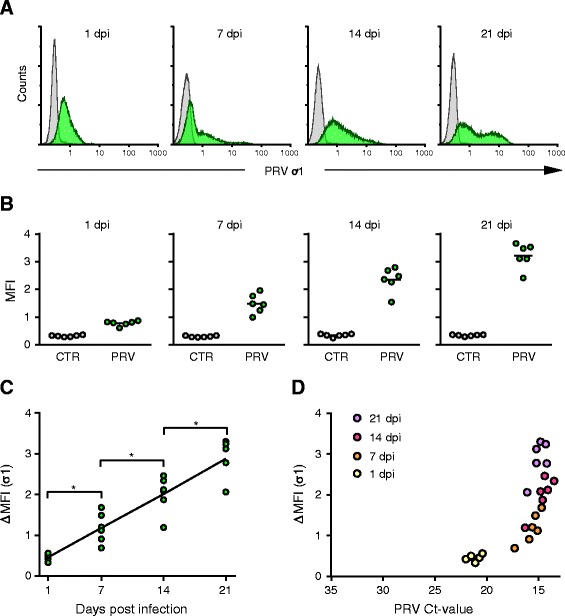
**Detection of PRV ơ1 protein by flow cytometry in the 1** ^**st**^ **passage. (A)** Results from intracellular staining for PRV ơ1 protein from PRV infected culture (green) and control culture (grey) at 1, 7, 14 and 21 days post infection (dpi). The fluorescence intensity (PRV ơ1) is shown on the X-axis and cell count on the y-axis counting 30 000 cells per sample. The results of one individual are presented to illustrate the staining pattern. **(B)** Plot of the arithmetic mean fluorescence intensity (MFI) for all PRV infected (green) and control (grey) cultures (*n* = 6) at each time point. **(C)** The amount of PRV ơ1 protein detected presented by ΔMFI (MFI Infected – MFI Control) at 1, 7, 14 and 21 dpi. Data were analyzed using Wilcoxon matched pairs signed rank test. *p < 0.05. **(D)** The correlation between the amount of PRV nucleic acid and PRV ơ1 protein detected during the cultivation is illustrated by plotting PRV Ct-values (X-axis) against the ΔMFI for PRV ơ1 (Y-axis) color coding the different time points; 1 dpi (yellow), 7 dpi (orange), 14 dpi (red) and 21 dpi (purple).

The infected RBC were stained intracellularly with a PRV ơ1-antibody and analyzed by flow cytometry to determine the level of viral protein during the first passage. At 1 dpi the RBC population observed in the PRV-infected cultures had shifted to the right compared to the uninfected controls (Figure 2A; 1 dpi), indicating cell association of PRV. At 7 dpi the main population partly overlapped the control culture, but a distinct right shoulder, constituting RBC with higher PRV ơ1 content, was evident in infected cultures (Figure 2A; 7 dpi). At 14 dpi a broader right shoulder was observed, and the entire PRV infected population had shifted to the right indicating a general increased amount of PRV ơ1 protein (Figure 2A; 14 dpi). At 21 dpi the PRV infected cells were separated into two populations; a PRV high and low positive population (Figure 2A; 21 dpi).

The pattern described above was observed in all infected cultures and was accompanied by elevated mean fluorescence intensity (MFI) (Figure 2B). The detection of PRV ơ1, as measured by ΔMFI, was significantly increased from each time point to the next during the first passage (*p* < 0.05) (Figure 2C). Whereas the main increase in viral load detected by RT-qPCR appeared during the early phases from 1 dpi to 7 dpi, the most prominent increase in viral protein was observed in later phases from 7 to 21 dpi (Figure 2D).

### Development of erythrocytic PRV inclusions

**Figure 3:**
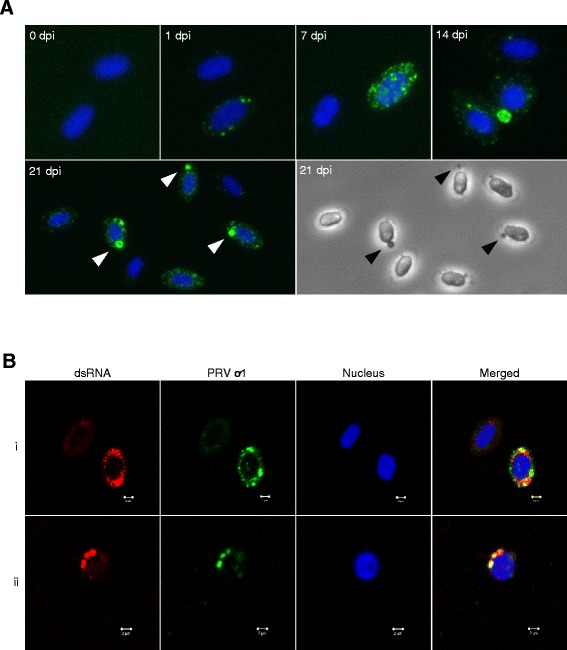
**Immunofluorescence and confocal microscopy of PRV infected RBC. (A)** Fluorescent labeling of the PRV ơ1-protein (green) in ex vivo infected RBC. The nuclei were stained with Hoechst (blue). Non-infected cells are shown at 0 dpi, and infected cells are shown at 1, 7 14 and 21 dpi. Large PRV inclusions at 21 dpi (marked by arrowheads) can also be observed in phase contrast. **(B)** Confocal images of ex vivo infected RBC at 21 dpi, stained with anti-dsRNA (red) and anti-PRV-ơ1 (green). The nuclei were stained with Hoechst (blue). dsRNA was detected within PRV inclusions, and either partly (i) or completely (ii) co-localized with PRV ơ1. Scale bar 2 μm.

PRV infected erythrocytes from passage one were inspected by immunofluorescence (IF) microscopy. At 1 dpi PRV positive staining was observed as small cytoplasmic granules within infected RBC (Figure 3A; 1 dpi). At 7 dpi a similar granular staining pattern was detected, including positive cells with increased fluorescence intensity (Figure 3A; 7 dpi). At 14 dpi positive cells with prominent large inclusion was observed. (Figure 3A; 14 dpi). The cellular morphology of the PRV infected cells varied, as both elongated ellipsoidal RBC and erythrocytes with rounded cell shape and nucleus was observed. At 21 dpi the number of cells with large inclusions was numerous (Figure 3A; 21 dpi). The large inclusions were also visible in phase contrast and co-localized with the immunofluorescent staining of PRV. The staining pattern observed by IF, i.e. increasing amounts of PRV ơ1, was in line with the flow cytometry observations.

We showed that the erythrocytic inclusions contained both dsRNA and PRV ơ1 protein by double staining of smears of infected RBC. The dsRNA staining partly or completely co-localized with the PRV ơ1 as observed by confocal imaging (Figure 3B). Fixation with methanol was necessary for detection of dsRNA in the co-lolcalization experiments. This fixation technique reduced the signal to noise ratio compared to the method used for flow cytometry and standard immunofluorescence microscopy (Figure 3A).

### Ex vivo infected RBC elicit an antiviral immune response

**Figure 4:**
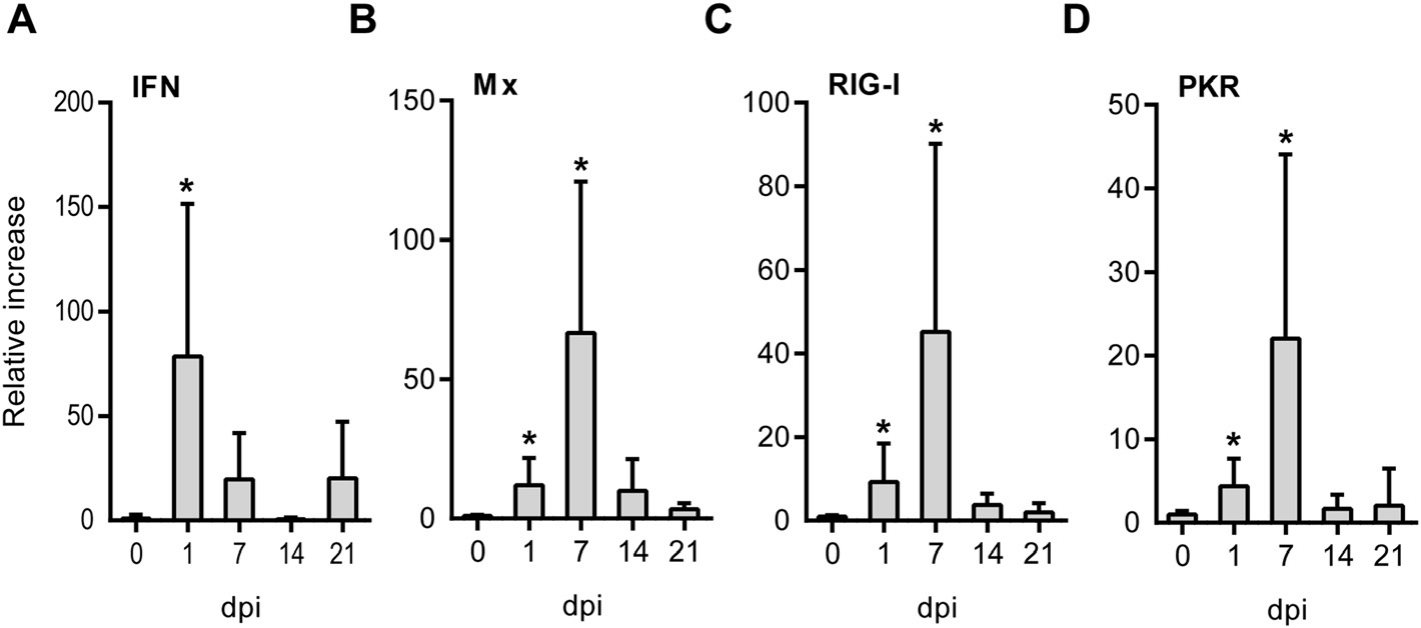
**Erythrocytic antiviral responses to PRV infection.** Expression of genes involved in antiviral responses was measured by RT-qPCR. The expression levels in infected RBC relative to the paired non-infected controls were calculated for each sample (*n* = 6) at 1, 7, 12 and 21 dpi. The relative increase (and SD) for IFNα **(A)**, Mx **(B)**, RIG-I **(C)** and PKR **(D)** is shown. Data were analyzed using Wilcoxon matched pairs signed rank test. *p < 0.05.

In order to determine if the PRV infected RBC responded to PRV infection by antiviral gene expression, the mRNA levels of IFN-α, Mx, RIG-I and PKR were analyzed. IFN-α was significantly up-regulated at 1 dpi (Figure 4A). A significant up-regulation at 1 and 7 dpi was observed for Mx (Figure 4B), RIG-I (Figure 4C) and PKR (Figure 4D). These latter three genes followed a similar expression pattern with peak expression detected at 7 dpi, before the expression levels returned to baseline.

### Successful passage of PRV

**Figure 5:**
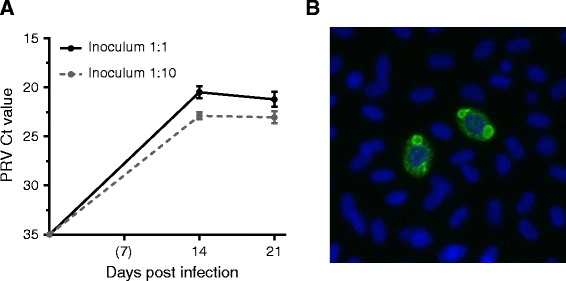
**RT-qPCR and PRV ơ1 staining from the 2** ^**nd**^ **passage. (A)** Mean PRV Ct value (and SD) detected by RT-qPCR in red blood cells (RBC) at 14 and 21 dpi (*n* = 6) during the second passage. Results are shown for cultures infected with undiluted and diluted (1:10) inoculum from the 1st passage. **(B)** Immunofluorescence microscopy picture showing the PRV ơ1-protein (green) in cells at 14 dpi infected by undiluted inoculum. Cell nuclei stained with Hoechst (blue).

Tenfold dilutions of the supernatant from passage 1 were used as inoculums in passage 2. The undiluted and 10-fold diluted inoculums were measured to Ct values of 27.0 and 31.6 respectively. Following inoculation there was an increase in viral load from 1 dpi until 14 dpi, and similar to passage 1 the increase leveled off thereafter (Figure 5A). The peak viral load was significantly higher in RBC given the undiluted compared to the 10 fold diluted inoculum at 14 dpi (Ct 20.5 ± 0.6 and Ct 22.9 ± 0.3 respectively) and 21 dpi (Ct 21.2 ± 0.7 and Ct 23.0 ± 0.6 respectively) (*p* < 0.05). In line with passage 1, PRV ơ1 positive RBC were observed at 14 and 21 dpi by IF microscopy (Figure 5B). However, the number of positive cells was much lower, and the PRV positive population was below the detection level for flow cytometry (data not shown).

## Discussion

In this study we demonstrated that PRV replicates in Atlantic salmon erythrocytes ex vivo. PRV obtained from infected fish were passaged to naïve erythrocytes. A significant increase in viral load was detected and viral factories containing PRV proteins and dsRNA was formed within infected RBC. Supernatant from the first passage was successfully passaged to naïve erythrocytes. In addition, we demonstrated that ex vivo infected RBC generated an innate antiviral immune response.

During the first passage, a significant increase in PRV load in the RBC fraction was observed by RT-qPCR and flow cytometry. At 1 dpi the mean Ct-value was relatively low, indicating that the added PRV efficiently associated with RBC. The results of flow cytometry corroborated this as the entire RBC population shifted to the right, suggesting that all RBC were PRV positive at this time point. The increase in the amount of viral RNA was most prominent in the early phase and plateaued at 14 dpi. The peak Ct-values were similar to those observed in previous in vivo challenge trials [12]. On the other hand, the viral load as detected by flow cytometry increased until 21 dpi, indicating a continuous production of PRV proteins within infected RBC. During ex vivo cultivation, PRV infected RBC formed two distinct populations observed by flow cytometry. This is similar to that observed during in vivo challenge experiments in which low and high PRV positive RBC populations are found during peak PRV infection [12].

The amount of PRV protein detected by flow cytometry were in line with the PRV staining observed by immunofluorescence microscopy. The PRV staining was observed as cytoplasmic inclusions similar to those observed during in vivo studies [12], and developed over time from small numerous granules to large distinct inclusions. At 14 dpi and 21 dpi, many RBC with one or two very large inclusions were observed, probably reflecting the high PRV positive RBC population. The inclusions were shown to contain both dsRNA and PRV ơ1 protein, although the degree of co-localization varied between cells. For MRV, the viral replication process takes place in cytoplasmic inclusions called viral factories [16]. These viral factories are shown to contain large amounts of both viral proteins and dsRNA and are essential for successful replication [16,40]. The globular inclusions detected in PRV infected RBC resembles the shape of the viral factories observed in MRV type 3 Dearing (T3D) infected cells [12,17,18]. Thus, both content and morphology of PRV inclusions are similar to the viral factories of orthoreoviruses.

A significant increase in PRV load was observed in the supernatant, which successfully passaged PRV to naïve erythrocytes. This indicates that infectious viral progeny is produced during the ex vivo cultivation. RBC with PRV inclusions were observed in the second passage, however, the number of positive cells was low and below detection level for flow cytometry. In previous in vivo experiments, the threshold of detection by flow cytometry appeared to be corresponding to a Ct-value of about 20 [12], thus slightly below the level obtained in the second passage. The viral titer of the inoculum was significantly higher in passage 1 (Ct-value 22.3) compared to passage 2 (Ct-value 27.0). This could explain the difference in virus outcome between the passages.

The amount of PRV within infected RBC was substantial; however, it was not accompanied by cell lysis (data not shown). The low, but increasing viral load detected in the supernatant suggests a slow but steady release of virus. This correlates well with in vivo observations where neither a PRV infection or an HSMI outbreak is associated with anemia [15]. However, HSMI is associated with circulatory disturbances, and a swollen spleen with high viral load is a common finding [12,41]. Orthoreovirus infections commonly results in cell lysis and release of progeny particles is thought to follow cell death and disruption of the plasma membrane [10]. However, non-lytic persistent infections have also been demonstrated in cell cultures [42-46]. The release mechanism of PRV remains to be shown. Although the present study suggests salmon RBC can tolerate high amounts of PRV, it is not known how it affects other important erythrocyte functions, such as oxygen transport.

Viral replication in erythrocytes is not well documented in the literature. Mature mammalian erythrocytes are not nucleated and thus not able to support a viral replication [47]. However, it is demonstrated that MRV can bind erythrocytes by the σ1 protein [48,49], and amino acid residues involved in this binding are partly conserved in the PRV σ1 protein [7,9]. In contrast to mammalian RBC, piscine erythrocytes are nucleated and retain ribosomes and a number of organelles while in circulation, thus competent to perform transcription and protein synthesis [27,30]. Differences in the content of PRV protein in RBC were observed during the present ex vivo infection and in previous in vivo experiments, indicating that the susceptibility of RBC varies [12]. During maturation and ageing of erythrocytes there is an inverse relationship between loss of cytoplasmic organelles and increase in hemoglobin content [50,51] accompanied by a reduced aerobic metabolic rate [52]. Both total RNA content and protein synthesis is higher in young erythrocytes [28]. It is a possibility that the putative differences in PRV susceptibility of erythrocytes is correlated to maturation.

PRV is the first virus shown to replicate in salmonid erythrocytes. However, there have been several reports of different viral inclusions in salmonid erythrocytes [19-22]. The most commonly reported is erythrocytic inclusions body syndrome (EIBS) [23], which have been associated with a transient anemia in freshwater Pacific salmon [53]. In contrast, EIBS has not been linked to anemia in Atlantic salmon [54]. No specific viral agent has been characterized for these findings. Interestingly, EM images of PRV and PRV inclusions strongly resemble some of those reported for EIBS [12,24-26]. Blood smears collected from experimentally PRV infected fish have been shown to stain positive by pinacyanol chloride [12]; the most commonly used method in detection and characterization of EIBS. However, a report suggested the EIBS agent to be enveloped, whereas PRV is a reovirus which is non-enveloped [55]. The present study demonstrated that erythrocytic PRV inclusions contain dsRNA, whereas a previous report suggested the EIBS agent to be a single stranded RNA virus based on acridine orange staining [26]. Pinacyanol chlorid and acridine orange are not agent specific staining methods, in contrast to the dsRNA MAb and PRV σ1 specific antibody used in the present study. Whether EIBS represents one viral agent or several related or non-related viral agents is not known and should be sorted out.

Prior studies have shown that salmon erythrocytes can elicit antiviral responses to Poly(I:C) stimulation [30]. We observed that PRV infection ex vivo induced gene expression of IFNα, Mx, RIG-I and PKR, indicating the mobilization of an antiviral response. The production of Mx, which is an interferon target gene [56,57], indicate that interferon is also produced at the protein level following PRV infection. Expression of RIG-I and PKR in particular indicate that the induced response may specifically target dsRNA viruses. This response may have the ability to inhibit or moderate further PRV infection and replication in the RBC cultures, leading to suboptimal viral production, and indicating that viral propagation in RBC ex vivo can be optimized further.

RBC is a major target cell for PRV in vivo and thus the study of PRV infection in erythrocytes will be central to progress our understanding of PRV infection and its implications on Atlantic salmon health. The lack of available cell lines supporting PRV replication has restricted studies of PRV infection and pathogenesis. The present study demonstrated an ex vivo infection model using primary naïve erythrocytes, which could be used to propagate PRV. The advantage of using RBC would be that it closely mimics the in vivo situation; therefore, the ex vivo cultivation model will likely benefit future studies of PRV infection, replication and release from RBC. In addition, it could be used to study the antiviral immune response of erythrocytes in salmonid fish.

## Abbreviations

CCL: CC chemokine ligand
Ct: Cycle threshold
dpi: Days post infection
dsRNA: Double-stranded RNA
EIBS: Erythrocytic inclusion body syndrome
EPC: Epithelioma papulosum cyprinid
FHM: Fathead minnow
HSMI: Heart and skeletal muscle inflammation
IFN-α: Interferon alpha
ISAV: Infectious salmon anemia virus
MDA: Melanoma differentiation-associated
MFI: Mean fluorescence intensity
MRV: Mammalian orthoreovirus
MX: Myxovirus resistance
PBS: Phosphate-buffered saline
PRV: Piscine orthoreovirus
RBC: Red blood cells
RIG: Retinoic-acid inducible gene
RT-qPCR: Real-time quantitative polymerase chain reaction
T3D: Type 3 Dearing
TEM: Transmission electron microscopy
TLR: Toll-like receptor
